# Projecting excess emergency department visits and associated costs in Brisbane, Australia, under population growth and climate change scenarios

**DOI:** 10.1038/srep12860

**Published:** 2015-08-06

**Authors:** Ghasem (Sam) Toloo, Wenbiao Hu, Gerry FitzGerald, Peter Aitken, Shilu Tong

**Affiliations:** 1 Research Fellow, School of Public Health and Social Work, Queensland University of Technology, Victoria Park Road, Kelvin Grove, QLD 4059, Australia; 2 Associate Professor, School of Public Health and Social Work, Queensland University of Technology, Victoria Park Road, Kelvin Grove, QLD 4059, Australia; 3 Professor, School of Public Health and Social Work; Director, Centre for Disaster and Emergency Management, Queensland University of Technology, Victoria Park Road, Kelvin Grove, QLD 4059, Australia; 4 Medical Director, Retrieval Services and Counter Disaster Unit, Queensland Health, 147-163 Charlotte St, Brisbane, QLD 4000, Australia; 5 Professor & Leader, Ecosystem Change and Population Health Program, School of Public Health and Social Work, Queensland University of Technology, Victoria Park Road, Kelvin Grove, QLD 4059, Australia

## Abstract

The direct and indirect health effects of increasingly warmer temperatures are likely to further burden the already overcrowded hospital emergency departments (EDs). Using current trends and estimates in conjunction with future population growth and climate change scenarios, we show that the increased number of hot days in the future can have a considerable impact on EDs, adding to their workload and costs. The excess number of visits in 2030 is projected to range between 98–336 and 42–127 for younger and older groups, respectively. The excess costs in 2012–13 prices are estimated to range between AU$51,000–184,000 (0–64) and AU$27,000–84,000 (65+). By 2060, these estimates will increase to 229–2300 and 145–1188 at a cost of between AU$120,000–1,200,000 and AU$96,000–786,000 for the respective age groups. Improvements in climate change mitigation and adaptation measures are likely to generate synergistic health co-benefits and reduce the impact on frontline health services.

Increasing demand for Australian public hospital emergency departments (ED) is associated with crowding and adverse health outcomes^1^,^2^, and considerable costs. While many studies report factors such as population aging, extended models of care, patient’s characteristics (socio-demographic, health and cognitive status), and seasonal illnesses and pandemics (e.g. influenza outbreaks) as general explanations for the increase^3^, recent studies confirm the effects of heatwaves and high temperatures on ED utilisation^4^,^5^,^6^. Additionally, monitoring emergency and acute health effects of high temperatures can assist with preparing response plans and implementing early warning programs to minimise the risk^7^,^8^. There is an urgent need for a long-term and systematic approach to disaster preparedness, health care resilience and improving the surge capacity of the emergency health system^9^,^10^.

The Fifth Report by Intergovernmental Panel on Climate Change (IPCC) projects that the average global surface temperature will increase by up to 4 °C by 2100 relative to 1986–2005^11^. Unless actions are taken to adapt, the adverse heat-related health consequences will also increase, resulting in even higher demand for emergency health services. This in turn will be associated with extra costs, capacity constraints (e.g. physical space, access block), and pressures on medical and non-medical personnel. To inform the development of adaptive actions, it is necessary to assess the past trends, understand the current situation, generate future scenarios, project the future demand for ED services based on the best information available, and evaluate and revise the projections as new information becomes available.

Brisbane is the capital city of Queensland, Australia, with a subtropical climate ranging from hot and humid summers to mild dry winters. In 2012, the greater Brisbane area had nearly 2.2 million residents of whom 11% were aged 65+ years. With an annual population growth rate of 2.5% (3.3% for 65+ year old age group), Brisbane is the second fastest growing city in Australia^12^,^13^. Despite exposure to warm and humid climate, the population is not necessarily immune to adverse health effects of high temperatures. Recent studies have highlighted the elevated risk of mortality, morbidity, and hospital admissions associated with hot temperatures^14^,^15^,^16^. This study aims to assess the excess demand for ED services associated with high temperatures, and to project future excess demand under climate change and population growth scenarios in Brisbane.

## Methods

### Study context

**Figure 1:**
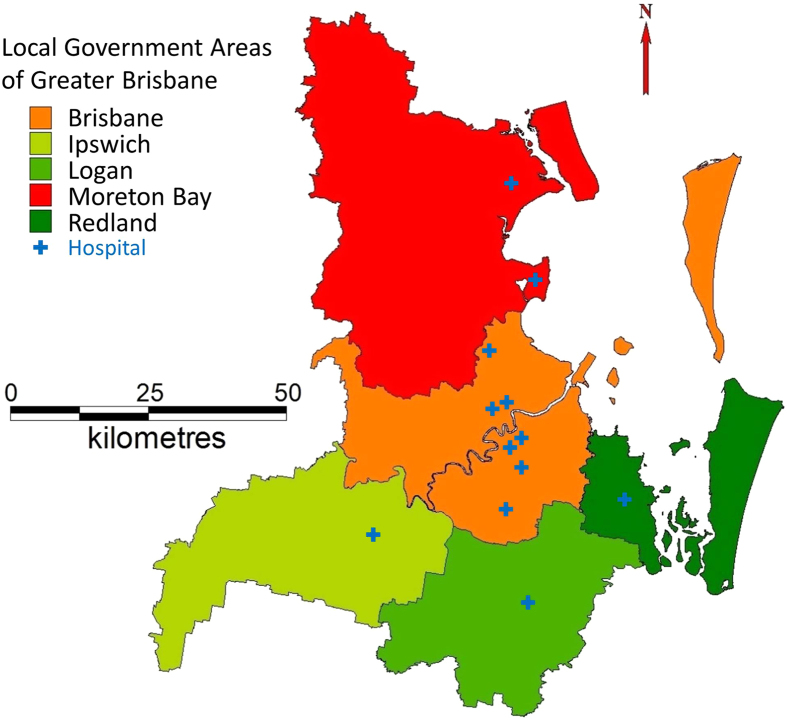
Map of study area. The map was created in MapInfo Pro 10.5 (Pitney Bowes Software) by Dr Xin Qi, former PhD student at Queensland University of Technology.

The epidemiological study was conducted in greater Brisbane area including Local Government Areas of Brisbane, Logan, Ipswich, Moreton Bay, and Redland (Fig. 1). Twelve public hospitals, comprising two specialist children’s, two tertiary, and eight major hospitals, provide emergency care in this area. These EDs received over 600,000 patients in 2012–13, at an estimated cost of over AU$300 million using 2012–13 prices^17^.

### Data

ED presentations to the 12 hospitals were provided by Queensland Health for summer seasons (December to February), 2000–2012. One ED was excluded because of incomplete data. The data contained arrival date, age, diagnostic code according to International Classification of Diseases (ICD) versions 9 and 10, triage category, and departure status. The reason for applying these two versions of ICD was that ICD-9 was used until 2006 in some EDs after which it was replaced with ICD-10. Applicable missing values for the variables were nil (arrival date), <0.01% (age), 1.3% (ICD), 0.2% (triage), and 13.3% (departure status). Due to high proportion of missing values for departure status, we randomly replaced these with values similar to the cases with known departure status based on their age group and triage category. This reduced the missing values to 0.3% for this variable. In total, 1,364,703 cases were included. However, cases with missing data were excluded pairwise from the analysis.

Climate data, from seven stations across Brisbane, including daily maximum temperature (Tmax) and relative humidity at 9 am and 3 pm were obtained from Bureau of Meteorology. Air quality data from nine stations including hourly amounts of ozone, particulate matter with <10 micrometres in diameter (PM_10_) and nitrogen dioxide (NO_2_) were provided by Department of Science, Information Technology, Innovation and the Arts. Data were averaged from all stations for each element.

To control for the potential effects of adaptation, we used air-conditioner penetration rate (i.e. percentage of households who owned this appliance) as a proxy indicator. Data for 2000–2010 were extracted from National Appliance and Equipment Energy Efficiency report^18^, and annual growth rate was used to estimate the rates for 2011 and 2012.

Population data including projections for 2030 and 2060 were downloaded from the Australian Bureau of Statistics’ website^13^,^19^. This population projection method takes into account a number of national and international trends including fertility, mortality, and migration. To simplify the analysis, the projections have been summarised into three major series: Series A represents the projected population if the growth is higher than the current level; Series B if the current growth rate continues; and Series C, if the growth rate declines^19^.

Projected number of hot summer days (Tmax ≥ 35 °C) based on Low and High greenhouse gas emission scenarios for Brisbane were extracted from the projections by Commonwealth Scientific and Industrial Research Organisation (CSIRO)^20^. Accordingly, the number of hot days is projected to range between 1–3 and 2–13 days in 2030 and 2060, respectively.

Costing data were downloaded from Independent Hospital Pricing Authority (IHPA)’s website for 2012–13^17^. As part of the *National Health Reform Act 2011*, IHPA was established by the Australian Government to provide independent advice about the efficient cost of public hospital services. IHPA’s primary role is to deliver a National Efficient Price (NEP) and price weights for various groups of activities in public hospitals, including EDs^21^. Since our data only contained the triage category and departure status, we adopted the pricing method called Urgency Disposition Groups (UDG). UDG categorises the data based on Australasian Triage Scale (Category 1 = Most urgent, Category 5 = Least urgent) and departure status (Admitted, Discharged). This creates ten groups (UDG-1 = Admitted, Triage-1 to UDG-10 = Discharged, Triage-5) plus two extra groups for patients who were “dead on arrival” or “did not wait”. Each UDG is assigned a price weight that when multiplied by the NEP, the cost for that group is determined. The price weights and NEPs are determined by IHPA as outlined in the *Pricing Framework for Australian Public Hospital Services*, and are updated annually^21^. In 2012–13, the NEP was set at AU$4808, and the price weights (PW) and estimated cost for each UDG were^17^:

1. UDG-1 = Admitted, Triage 1, PW: 0.2996, estimated cost: AU$1440.5;
2. UDG-2 = Admitted, Triage 2, PW: 0.2061, estimated cost: AU$990.9
3. UDG-3 = Admitted, Triage 3, PW: 0.1801, estimated cost: AU$865.9
4. UDG-4 = Admitted, Triage 4, PW: 0.1531, estimated cost: AU$736.1
5. UDG-5 = Admitted, Triage 5, PW: 0.1165, estimated cost: AU$560.1
6. UDG-6 = Non-Admitted, Triage 1, PW: 0.2203, estimated cost: AU$1059.2
7. UDG-7 = Non-Admitted, Triage 2, PW: 0.1475, estimated cost: AU$709.2
8. UDG-8 = Non-Admitted, Triage 3, PW: 0.1136, estimated cost: AU$546.2
9. UDG-9 = Non-Admitted, Triage 4, PW: 0.0768, estimated cost: AU$369.3
10. UDG-10 = Non-Admitted, Triage 5, PW: 0.0477, estimated cost: AU$229.3
11. UDG-11 = Did Not Wait, PW: 0.0353, estimated cost: AU$169.7
12. UDG-12 = Dead on Arrival, PW: 0.0440, estimated cost: AU$211.6

### Analysis

Age was split into 0–64 and 65+ years. ICD codes were recoded into four illness categories: Total; Non-external causes (NEC), External, and Heat-related illnesses (e.g. heatstroke, sunstroke and heat fatigue). NEC included codes 001–799 and V01–V91 (ICD9), and A00–R99 and Z00–Z99 (ICD10); External included codes 800–999 and E800–E999 (ICD9), and S00–Y98 (ICD10); and Heat-related illnesses included codes 992 and E900.0 (ICD9) and T67 and X30 (ICD10). Heat-related illnesses are a specific subgroup of external causes, and have been separately analysed here due to their overt association with exposure to external sources of heat. However, heat effects are often manifested as illnesses (e.g. cardiovascular, diabetes and renal complaints) exacerbated by heat^22^.

Generalised linear regression with Poisson link was used to assess the added effect of hot temperatures on ED visits. The model controlled for potential confounding effects of long-term trends, seasonality and weekday variations, air-conditioner penetration, relative humidity, ozone, PM_10_, and NO_2_. Population size was adjusted for by including an offset term in the models. Relative risks (RR) and 95% confidence intervals (CI) were calculated to assess the risk of ED visits for each illness–age group on hot versus non-hot days.

For projections, we calculated mean incidence rates of ED visits during study period on a non-hot day for each illness–age group (Base). We then applied the Base incidence rates to the projected populations (Series A, B and C) to estimate the expected number of ED visits in 2030 and 2060. Assuming the RRs would remain the same, the number of ED visits was multiplied by the relevant RR for each illness–age group to obtain the projected number of visits on hot days based on emission scenarios. We deducted the expected number of visits from the projected number to calculate the excess ED visits on hot days.

To estimate the costs, we analysed the distribution of ED visits during the Base year by triage category and departure status for each age group. We then multiplied the 2012–13 UDG weighted prices by the number of cases in each category. The average costs were AU$521 and AU$662 per person for age groups 0–64 and 65+ years, respectively. Assuming the same distribution would be applicable to the projection years, we multiplied the average costs by the number of excess visits in 2030 and 2060.

Statistical Package for the Social Sciences (SPSS) 21.0 was used for the analyses (IBM Software).

### Ethical approval

Ethics approvals for the use of health data were granted by Human Research Ethics committees at Queensland University of Technology and Queensland Health.

## Results

In total, 1083 days were included in the analysis, out of which 22 days (mean = 1.8 days for Base) were hot (Tmax ≥ 35 °C). Tmax ranged between 20.3 °C and 40.2 °C (mean = 29.8 ± 2.4) (Table 1).

Table 2 shows that 85.2% of the patients were 0–64 years old. NEC and external constituted 56.8% and 40.7% of the presentations (2.5% were non-specific or without a diagnosis, “did not wait” or “dead on arrival”). Heat-related illnesses formed <0.1% of the visits.

Total ED visits significantly increased on hot days (RR = 1.06, 95% CI: 1.05, 1.07). Age was positively associated with increased visits, for both younger (RR = 1.06, 95% CI: 1.05, 1.08) and older groups (RR = 1.9, 95% CI: 1.06, 1.13). The risk for NEC increased significantly in both age groups (RR = 1.08 vs. RR = 1.06). External causes increased significantly higher in the older group (RR = 1.18, 95% CI: 1.12, 1.25) than the younger (RR = 1.04, 95% CI: 1.02, 1.06). Heat-related visits also increased significantly among both the older (RR = 3.75, 95% CI: 2.44, 5.75) and younger groups (RR = 2.11, 95% CI: 1.51, 2.95).

**Figure 2:**
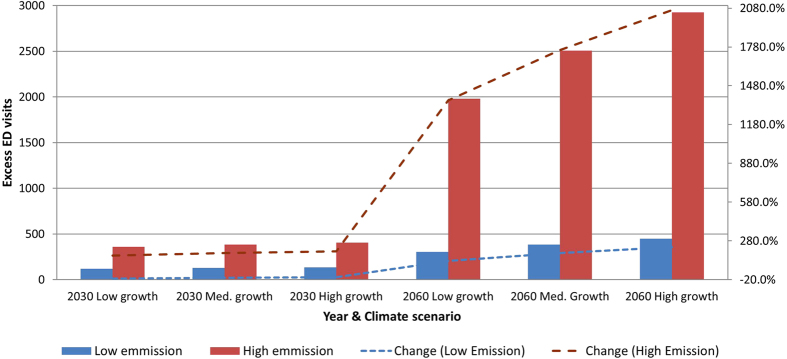
Projected number of excess ED visits on hot days (Tmax ≥ 35°C) and percentage of change compared to Base period, per Climate Change and Population Growth scenario.

In total, 135 excess visits were made on hot days during the Base year. Figure 2 shows that the number of excess ED visits in 2030 will range between 120 (low emission and population growth) and 405 (high emission and population growth). These represent a change of −11% and 200% compared to Base. By 2060, excess visits will range between 305 and 2925, respectively, representing increases of 125% and 2065%.

Table 3 shows that projected excess ED visits in 2030 will range between 98–336 and 42–127 in the younger and older groups, respectively. In 2060, the projected estimates are 229–2300 and 145–1188. In 2030, the excess NEC visits is projected to range between 69–238 and 19–58 for the younger and older groups, respectively. By 2060, these will be 162–1629 and 66–543. For external causes, excess visits in 2030 are projected between 27–93 and 23–71 for the two age groups. By 2060, these are estimated to range between 63–71 and 81–664.

Table 4 projects the costs of excess ED visits based on 2012–13 prices. In 2030, excess costs are projected to range between AU$51,000–184,000 for 0–64 years, and AU$27,000–84,000 for 65+ years age group. By 2060, these figures are estimated to range between AU$120,000–1,200,000 and AU$96,000–786,000 for the two groups, respectively.

## Discussion

Our projections show that ED presentations will increase considerably on hot days in the future under most population growth and climate change scenarios. The excess number of visits by older patients is estimated to grow twice as much as the younger group’s. The excess demand is estimated to add an extra cost of around AU$78,000–260,000 in 2030 and AU$215,000–1,985,000 in 2060, based on 2012–13 prices without adjusting for factors such as inflation rate.

This is, to our knowledge, the first study to project the long-term impacts and costs of climate change on ED services. Unlike mortality, little information is available on climate change impacts on morbidity and use of health and medical services in general, and ED services in particular^23^. This study complements the extant literature about the health effects of heat, and contributes new knowledge by quantifying the long-term impacts and costs. The findings have major implications for future research and policy in health service utilisation and management. EDs are under pressure from growth in demand^3^,^24^, and the added effect of high temperatures can impose further workload and financial constraints. This information can help policy-makers and health managers prepare the relevant infrastructure and improve policies and practices. Mitigation strategies and adaptation measures to reduce climate change risks can produce synergistic health co-benefits^25^. For instance, increasing green space can reduce heat island effect and encourage physical exercise which in turn benefits health. Projection studies shall provide the impetus and forum for improvements in risk prediction and reduction strategies which require a longer timeframe to establish.

This study also has some limitations. Assessment of climate change-related health effects involves uncertainties. Although we have based our projections on reliable scientific sources and assumptions about climate change and population growth^19^,^20^, realisation of the projections is not guaranteed as other factors can alter the outcomes. Drastic deterioration of air quality in the future, for instance, may interact with high temperatures and aggravate conditions such as respiratory and cardiovascular diseases^26^.

Global warming may also increase the number and intensity of events such as floods and bushfires which may affect people’s health, and compound the heat effects. Other factors such as the pattern of urbanization, the nature of the built environment, the development of early warning systems, the quality and accessibility of health care services, population health status, and many other factors will drive future vulnerability. Changes in adaptation may affect the projections. Although we included air-conditioner penetration as a proxy indicator, more detailed data are required, including actual use of air-conditioners, and other cooling methods (e.g. cooling shelters). Such data are not available and requires purpose-built surveys to complement existing data.

Furthermore, technological advances in reducing heat effects such as improved building designs and green space, lifestyle changes, and health care delivery advancements may alter the health effects of heat and hence the projections. We only analysed public hospitals’ data. Data from private hospitals and general practitioners were not available. Therefore, the overall impact and costs of heat are likely to be underestimated. Weather patterns and population growth may not be the only drivers of future visits to EDs as other factors such as affordability and accessibility of alternative healthcare services may affect the demand for EDs. And finally, today’s costs and prices may not necessarily reflect the future, as other factors such as technological advancements may affect these costing estimates.

The findings are overall consistent with current estimates of heat associated ED utilisation^4^,^5^,^6^. While, the overall risk increased by 6% on hot days, it was slightly higher among the elderly (RR = 1.09 vs. RR = 1.06). Comparatively, in Sydney, Australia, all-cause ED visits increased during heatwaves (RR=1.02; 95% CI: 1.01, 1.03), and was significantly higher among the 75+ years (RR = 1.08; 95% CI: 1.04, 1.11) than the <75 years (RR = 1.01; 95% CI: 1.00, 1.02)^4^. Another study in California, USA, reported increased all-cause ED visits during heatwaves (RR = 1.03; 95% CI: 1.02, 1.04), with no differences among age groups^6^. Differences in the risk magnitude across studies may reflect a number of factors such as study design and population characteristics. For instance, the mentioned studies compared heatwave periods with reference periods, while we compared hot versus non-hot days which we needed to use to be able to make projections. Population health and preparedness may also explain differences between studies and locations.

When we stratified the results by illness and age groups, more prominent differences emerged. The risk for NEC visits increased by 8% and 6% among the younger and older age groups, respectively. This is in contrast with findings from a hospital in Adelaide, Australia, that the absolute number of ED visits for a number of internal illnesses increased by 14% and 21% among age groups 15–64 and 65+ years during a 2009 heatwave compared to the average number of visits before and after the event^5^. The differing patterns between the findings of our study and Adelaide may be partly explained by differences in settings (population and climate) and designs (11 vs. 1 hospital; hot days vs. heatwave period), as mentioned above, and the number of illnesses analysed (all NEC visits vs. a few specific causes).

Of particular note in our findings is the 18% increased risk of external cause visits by older versus 4% increase by younger groups. We did not find any other study to compare. Considering heat-related illnesses, as a specific sub-set of external causes, the risk more than doubled and tripled for the two age groups. The Sydney study reported that acute heat-related ED visits increased approximately eight times from an average of 13.5 cases during the reference period to 104 cases during heatwaves^4^. The RR for heat-related illnesses in California was 6.3 (95% CI: 5.7, 7.0)^6^. The Adelaide study reported an absolute increase from average 0.5 and 2 cases during the reference period to 21 and 42 cases during the heatwave among younger and older patients, respectively^5^. Again, differences in study designs and population characteristics may explain the differences in results.

Assuming that current estimates will remain applicable, total excess ED visits on hot days will double by 2030 and increase by up to 21 times in 2060. We were unable to locate comparable studies. Existing forecasts focus on short-term and daily visits^27^, which is useful for day-to-day management of the facilities and operations, but do not factor in long-term effects of climate change and population growth. Our findings corroborate Australian and international projections that unless adaptation and mitigation actions are implemented, the health impacts of climate change will increase^28^,^29^,^30^.

Our estimates show that heat impacts on ED visits will rise substantially under high emission scenarios, particularly in 2060 as the number of hot days is projected to increase considerably. The projected excess ED visits are likely very conservative as they only reflect same-day effects of hot temperatures and do not include potential lagged effects. Current studies show that heatwave effects on health and service utilisation are usually immediate to short-term^4^,^15^,^31^. However, in the absence of projections on frequency and length of heatwaves in 2030 and 2060, we were unable to assess their immediate and lagged effects.

The findings show that heat effects vary across different age and illness categories. NEC covers a wide range of illnesses including infectious, cardiovascular, respiratory, renal, diabetic, and nephritic diseases. External causes include conditions such as injuries and poisoning, heat stress, and dehydration. Some of these conditions are exacerbated by exposure to heat, particularly among the elderly and children, leading to elevated mortality, hospital admissions and emergency visits^6^,^15^, and are likely to increase because of rising temperatures in the future^23^,^28^,^29^,^30^. Further research is required to determine how these (or emerging) diseases or changing patterns of illnesses as a result of climate warming may specifically contribute to demand for ED services in the future.

As heat-related health impacts are highly location- and population-specific, the findings of this study may not be directly generalisable to other locations and populations. Any attempt to reproduce the study in another location should take into account the climate (e.g. tropical, arid), community preparedness (e.g. early warning systems, cooling shelters), population characteristics (e.g. aging, poverty and disadvantage), and individual circumstances (e.g. age, work conditions, health status, socioeconomic status).

## Additional Information

**How to cite this article**: Toloo, G. (S.) *et al.* Projecting excess emergency department visits and associated costs in Brisbane, Australia, under population growth and climate change scenarios. *Sci. Rep.* **5**, 12860; doi: 10.1038/srep12860 (2015).

## Figures and Tables

**Table 1:** Descriptive characteristics of environmental variables, Dec–Feb, 2000–2012.

	Mean (95% CI)	Standard Deviation	Minimum	Maximum	Median
Aircon. Penetration (%)	53.2 (46.8, 59.7)	11.9	29.4	63.8	59.3
Tmax (°C)	29.8 (29.7, 30.0)	2.4	20.3	40.2	29.7
Relative humidity (%)	59.9 (59.2, 60.6)	11.9	20.8	95.8	58.2
O_3_ (ppm)	0.02 (0.02, 0.02)	0.01	0.0	0.04	0.01
PM_10_ (μg/m3)	18.4 (18.0, 18.8)	6.7	4.7	81.3	17.6
NO_2_ (ppm)	<0.01 (0.00, 0.00)	0.0	0.0	0.01	0.0

**Table 2:** ED visits, Incidence Rates, and Relative Risks on hot (Tmax ≥ 35 °C) versus non-hot days: Dec–Feb, 2000–2012.

Illness & Age	No. of ED visits (% on hot days)	Mean daily (±SD†)	Incidence rate (per 100,000)	Relative risk* (hot vs. non-hot)	95% CI
Total	1,364,703 (2.0)	1260.1 (170·5)	67.9	1.06	(1.05, 1.07)
All-cause 0–64	1,162,416 (2.0)	1073.3 (149·1)	64.6	1.06	(1.05, 1.08)
All-cause 65+	202,287 (2.1)	186.8 (32·2)	90.3	1.09	(1.06, 1.13)
NEC 0–64	633,455 (2.0)	584.9 (91·3)	35.1	1.08	(1.06, 1.10)
NEC 65+	142,056 (2.0)	131.2 (24·8)	63.3	1.06	(1.02, 1.10)
External 0–64	498,138 (2.0)	460.0 (78·6)	27.7	1.04	(1.02, 1.06)
External 65+	56,887 (2.2)	52.5 (13·5)	25.4	1.18	(1.12, 1.25)
Heat 0–64	438 (13.2)	1.6 (1·1)	<0.1	2.11	(1.51, 2.95)
Heat 65+	190 (27.9)	1.5 (2·1)	0.1	3.75	(2.44, 5.75)

^*^Controlled for long-term annual, monthly and weekday variations, air conditioner penetration, relative humidity, O_3_, PM_10_, NO_2_, and adjusted for population size.

^†^SD: Standard Deviation.

**Table 3:** Projected number of excess ED visits on hot days (Tmax ≥ 35 °C) per Climate and Population Scenario.

Illness & Age	Base (1.8 hot days)	Projection Year & Climate Scenario	Projected Hot days	Population Growth Scenario		
				C	B	A
All-cause 0–64	117.7	2030 Low emission	1	97.9	106.2	112.1
		2030 High emission	3	293.6	318.6	336.4
		2060 Low emission	2	229.4	305.0	353.9
		2060 High emission	13	1491.2	1982.5	2300.2
All-cause 65+	30.8	2030 Low emission	1	41.7	41.6	42.5
		2030 High emission	3	125.0	124.7	127.4
		2060 Low emission	2	145.3	153.6	182.8
		2060 High emission	13	944.2	998.5	1188.1
NEC 0–64	83.4	2030 Low emission	1	69.3	75.2	79.4
		2030 High emission	3	208.0	225.6	238.3
		2060 Low emission	2	162.5	216.0	250.6
		2060 High emission	13	1056.2	1404.2	1629.2
NEC 65+	14.1	2030 Low emission	1	19.1	19.0	19.4
		2030 High emission	3	57.2	57.0	58.3
		2060 Low emission	2	66.4	70.3	83.6
		2060 High emission	13	431.8	456.7	543.4
External 0–64	32.5	2030 Low emission	1	27.0	29.3	30.9
		2030 High emission	3	81.0	87.9	92.8
		2060 Low emission	2	63.3	84.1	97.6
		2060 High emission	13	411.3	546.8	634.5
External 65+	17.2	2030 Low emission	1	23.3	23.2	23.7
		2030 High emission	3	69.8	69.7	71.2
		2060 Low emission	2	81.1	85.8	102.1
		2060 High emission	13	527.4	557.8	663.7
Heat 0–64	0.7	2030 Low emission	1	0.6	0.6	0.7
		2030 High emission	3	1.8	1.9	2.0
		2060 Low emission	2	1.4	1.9	2.1
		2060 High emission	13	9.0	12.0	14.0
Heat 65+	0.6	2030 Low emission	1	0.9	0.9	0.9
		2030 High emission	3	2.6	2.6	2.6
		2060 Low emission	2	3.0	3.2	3.8
		2060 High emission	13	19.5	20.7	24.6

**Table 4:** Projected Costs of Excess ED visits on hot days (Tmax ≥ 35 °C) per Climate Change and Population Growth scenario (AU$, 2012–13 prices).

Illness & Age	Base (1.8 hot days)	Projection Year & Climate Scenario	Projected hot days	Population Growth Scenario			% Change compared to Base		
				C	B	A	C	B	A
All-cause 0–64	61,330	2030 Low emission	1	50,997	55,323	58,424	−16.8	−9.8	−4.7
		2030 High emission	3	152,990	165,969	175,272	149.5	170.6	185.8
		2060 Low emission	2	119,527	158,903	184,366	94.9	159.1	200.6
		2060 High emission	13	776,926	1,032,867	1,198,380	1166.8	1584.1	1854.0
All−cause 65+	20,442	2030 Low emission	1	27,581	27,518	28,112	35.1	34.7	37.7
		2030 High emission	3	82,743	82,553	84,335	305.2	304.2	313.0
		2060 Low emission	2	96,162	101,696	121,001	370.9	398.0	492.5
		2060 High emission	13	625,052	661,023	786,506	2960.6	3136.8	3751.2
TOTAL	81,752	2030 Low emission	1	78,578	82,841	86,536	1.0	−3.8	−9.8
		2030 High emission	3	235,733	248,522	259,607	203.0	188.7	170.5
		2060 Low emission	2	215,689	260,598	305,367	240.1	191.4	132.2
		2060 High emission	13	1,401,978	1,693,890	1,984,887	2110.5	1794.1	1409.4
